# Staphylococcal Scalded Skin Syndrome in Neonate

**DOI:** 10.1155/2015/901968

**Published:** 2015-06-08

**Authors:** K. Kouakou, M. E. Dainguy, K. Kassi

**Affiliations:** ^1^Department of Pediatrics, Training and Research Unit of Medical Sciences, Felix Houphouët Boigny University of Abidjan, Côte d'Ivoire; ^2^Department of Dermatology and Infectiology, Training and Research Unit of Medical Sciences, Felix Houphouët Boigny University of Abidjan, BP 5151, Abidjan 21, Côte d'Ivoire

## Abstract

We described a case of Staphylococcal Scalded Skin Syndrome in infant age of 21 days by discussing clinical and management issues. This newborn presented large erythematous, eroded, and oozing areas covered by epidermal skin flap. The average surface of cutaneous unsticking on admission was 31.35% of body surface area corresponding to lesions of superficial second-degree burns. An important biological inflammatory syndrome including positive C-reactive protein was found. Under treatment, erythroderma decreased within 7 to 10 days and the newborn was completely healed after 3 weeks of followup, with the disappearance of the inflammatory syndrome and total body surface restored. This clinical case report showed that SSSS remains a major dermatological problem in neonates. Therefore, its diagnosis should be made without doubt and its care should start earlier in a neonate emergency unit in order to have good prognosis. And the rigorous “search and destroy” policy based on screening of staff and patients and isolation of identified patients advocated in the United Kingdom should be applied in neonate units in Côte d'Ivoire.

## 1. Introduction

Staphylococcal Scalded Skin Syndrome (SSSS) or acute staphylococcal epidermolysis is an exfoliative skin disease and a toxin mediated staphylococcal infections affecting mostly neonates and adolescents and it is rare in adults [1, 2]. Currently, the incidence of this disease is increasing in all ages. Its resistance to conventional antibiotic treatment is also a new reality. Prognosis is mostly favourable and skin lesions healed without scarring [3]. We describe a case of a newborn of 21 days of age with SSSS and discuss relevant pathology, clinical issue, and management.

## 2. Case Report

A newborn was hospitalized for erythroderma. The disease started with a sore throat and conjunctivitis. Within 48 hours, the newborn developed a fever and tender erythema which progresses to generalized erythematous skin lesions mostly seen in the axillary and groin areas. It was associated with formation of large fragile-roofed superficial blisters which rupture on the slightest pressure leading to extended areas of denuded and eroded skin. The Nikolski (easy separation of skin layers upon application of horizontal, tangential pressure to the skin) sign was present.

The medical history showed that her mother got pregnant two times. Her blood group was AB positive. The clinical examination during mother's pregnancy was normal out of the hemoglobin type which was abnormal (type AC). HIV test was negative. Vaccination for tetanus and hepatitis B was up to date. Her mother had not any blistering disease history. Drugs taken during mother's pregnancy were “Tanakan, Folifer, and Fansidar tablets,” used at the 26th and the 32nd weeks of pregnancy according to the national program for malaria control in Côte d'Ivoire.

We found a history of traditional medicines use from the 3rd month of pregnancy to vaginal delivery. This delivery was normal under epidural anesthesia with marcaine at the 40th week of pregnancy. There was no family history of similar skin lesions.

In the birth, it was a female newborn weighing 3.7 kilograms (Kg) and of the size 45 centimeters (cm). Cranial perimeter was estimated to be 33 cm and the APGAR score was estimated to be 8 at the first minute and 9 at the 5th minute.

Its clinical examination on admission revealed no fever with 36,2°C of temperature, and the respiratory frequency was 45 cycles/minute. The heart rate was 120 beatings/minute, and integuments were colored.

**Figure 1:**
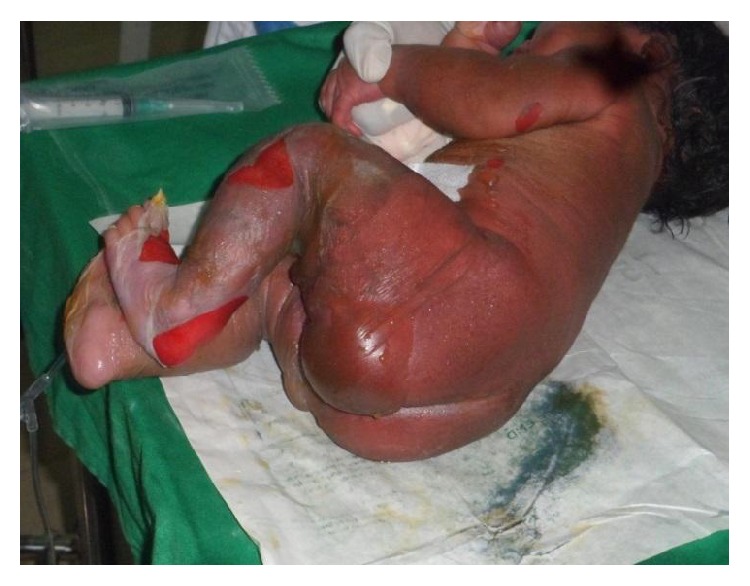
Staphylococcal Scalded Skin Syndrome in a newborn with generalized bullous epidermolysis.

The examination of the skin and the mucous membranes highlighted the skin peeling and widespread blisters prevailing in the anterior and posterior parts of the lower limb relying on an erythematous basis. The epidermal necrolysis has quickly extended to the bottom and to the trunk during hospitalization. There appear large erythematous, eroded, and oozing areas covered by epidermal skin flap (Figure 1). Mucous membranes were intact. The genital examination showed unsticking lesions on the big and small lips of the vagina which bled when in contact. Other body system examinations were normal. The average surface of cutaneous unsticking on admission was 31.35% of the body surface area corresponding to the lesions of superficial second-degree burns.

We found an important biological inflammatory syndrome including positive C-reactive protein.

Three differential diagnoses were evoked with these clinical manifestations: (1) toxic epidermal necrosis drug induced (Lyell syndrome), (2) Staphylococcal Scalded Skin Syndrome (SSSS), and (3) Staphylococcal Shock.

**Figure 2:**
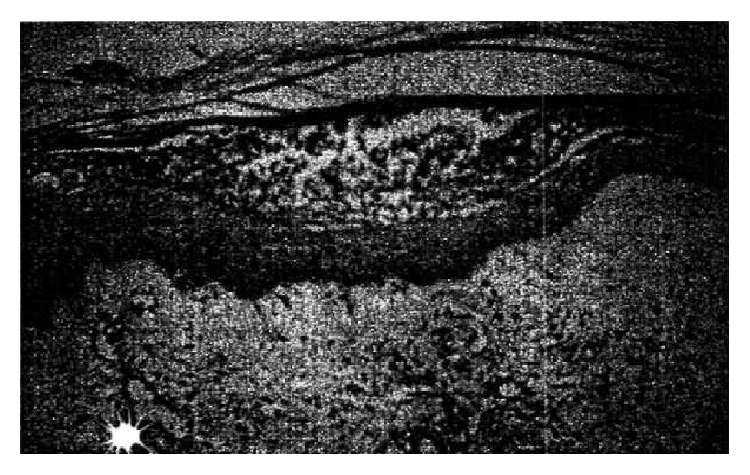
Superficial epidermolysis of the granular layer.

The skin biopsy has shown superficial intraepidermal split into the granular layer (Figure 2) associated with little inflammatory infiltrate in the superficial dermal layer and no necrosis. This aspect characterizes SSSS.

Bacteriological examinations of urine, skin, and vaginal swabs were negative. The conjunctivae and nasopharyngeal cultures and the blood culture were positive to* Staphylococcus aureus*. In some, the diagnosis is done, based on (1) clinical finding of superficial blisters, (2) intraepidermal split on histology, and (3) demonstration of staphylococcal infection. In addition, the biological blood check did not find any abnormalities such as anemia and renal impairment. Immunofluorescence was not performed due to the lack of material in our setting.

The newborn was treated by a double antibiotic therapy combining ceftriaxone and aminoside at the neonate emergency unit. We added an important rehydration, antipain, and skin care with eosin liquid 1%. The treatment was effective and the outcome was rapidly favourable. The erythroderma decreased within 7 to 10 days and the newborn was completely healed after 3 weeks of followup, with disappearance of the inflammatory syndrome and total body surface restored.

## 3. Discussion

Generally, SSSS is regarded as mild disease, but in neonate and immunocompromised patient it is serious and occasionally fatal and the exfoliative toxins produced by* Staphylococcus aureus* are considered to be the pathogenetic agent in SSSS [4]. These exfoliative toxins are also responsible for causing bullous impetigo. It appears to be a relationship between the disease extent, the amount of toxin produced, and whether the toxin is released locally or systemically [3]. There are two exfoliative toxins that are identified, exotoxin A, the most produced one, and the exotoxin B. Most strains of* Staphylococcus aureus* isolated from patient suffering from SSSS belong to phage group II (about 80%) [3, 5]. These toxins have exquisite specificity in causing loss of desmosome-mediated cell adhesion within the superficial epidermis only [6]. When the toxins are released into the blood stream, the lack of protective antitoxin antibody in neonates allows the toxins to reach the epidermis where they act locally to produce the characteristic skin lesions [7]. These human exfoliative toxin antibodies which have neutralizing properties decrease from 0 to 3 months [8]. This could explain the severity of our case who is 21 days of age, and, unfortunately, we could not identify exfoliative toxin secretion and its type.

These toxins cause histopathologically a subcorneal split along the granular cell layer, resulting from intra-epidermal acantholysis, as we observed in our case where we found little dermal inflammatory cell and no cell necrosis.

This is similar to that found in bullous impetigo, but in bullous impetigo there is pronounced inflammatory cell infiltrate consisting mostly in neutrophils [3].

Thus, in our case, 2 risk factors were identified, the lack of the newborn's immune system and the use of traditional products during the pregnancy and delivery periods by her mother, which might favour the occurrence of the disease.

All these pathogenesis characteristics allow us to understand the clinical manifestations of SSSS particularly in neonate.

SSSS has usually a swift onset of painful, tender, and red skin accentuated in flexural and periorificial areas. After 24 to 48 hours, flaccid blisters and erosions develop and large areas of the overlying epidermis loosen and peel like a scald which can be extended [9]. In our case, the disease starts with the inflammation of the conjunctivae (conjunctivitis) which is a* Staphylococcus* commensal site like umbilicus and axilla.

The diagnosis is usually made on clinical ground [9]; it relies mainly on the recognition of the characteristic appearance of the rash with fever. But it is important to swab the skin, the orificial areas, and the mucus membranes for bacterial confirmation and to identify the primary focus infection and screening for* Staphylococcus aureus* carriage, as we performed in our case. The skin biopsy often shows a superficial intraepidermal split into the granular layer associated with little inflammatory infiltrate in the superficial dermal layer without necrosis as we found in our case. This diagnosis is made in our case based on (1) clinical finding of superficial blisters, (2) intraepidermal split on histology, and (3) demonstration of staphylococcal infection.

### 3.1. Treatment

Antistaphylococcal antibiotics, temperature regulation, maintaining fluid and electrolyte balance, nutritional management, and skin care form the basics of treatment [3].

These antibiotics represent one of the main pillars of SSSS treatment, but the growing concerns of the resistant strains of staphylococci anti-ETA and anti-ETB might be the future challenges.

In fact, resistance was observed for some antibiotics: 5% for gentamicin, 7% for tetracycline, and 2% for chloramphenicol, whereas there were no strains resistant to methicillin, cephalothin, cephalexin, and vancomycin [4].

In practice, blisters should be left intact because it helps to reduce further trauma to the skin. Topical antibiotics or antiseptic eye ointment is also helpful to manage the conjunctivitis. In the best case, patients should be managed in the pediatric intensive care unit and consideration needs to be given to mattress requirement, pain management, temperature regulation, fluid management (rehydration), nutrition, and skin care. Corticosteroids are contraindicated with the worsening of the disease [3]. Appropriate intravenous antibiotics against penicillin-resistant staphylococci should be used such as methicillin and flucloxacillin and the use of intravenous fluid management and analgesia in case of oral intake is reduced because of the perioral lesions [7]. In our case, we used a double antibiotic therapy combining ceftriaxone and aminoside at the neonate emergency unit. We added important rehydration, antipain, and skin care with eosin liquid of 1%. The treatment was effective and the newborn was completely healed after 3 weeks of followup, with the disappearance of the inflammatory syndrome and total body surface restored.

### 3.2. Followup

The prognosis of SSSS in childhood is mostly favourable. The mortality rate is approximately 4% and it is associated with extensive skin involvement [3]. In our case, under treatment, erythroderma decreased within 7 to 10 days and the newborn was completely healed after 3 weeks of followup, with the disappearance of the inflammatory syndrome and total body surface restored.

### 3.3. Prevention

As asymptomatic nasal carriage of staphylococci aureus is an important source of infection in neonates, strict control measures should be taken such as isolation of infected patients, barrier nursing, and antiseptic hand washing by both staff and visitor to the unit. The rigorous “search and destroy” policy based on screening of staff and patients and isolation of identified patients that is now being increasingly advocated in United Kingdom [9] should be applied in neonate units in Côte d'Ivoire.

## 4. Conclusion

While most cases of SSSS are easily treated, it remains an emergency case and a potential fatal condition in neonate. Its diagnosis should be made without doubt and its care should start early in neonate emergency unit in order to have good prognosis.
